# Correct Identification of *Ochrobactrum anthropi* From Blood Culture Using *16rRNA Sequencing*: A First Case Report in an Immunocompromised Patient in Mexico

**DOI:** 10.3389/fmed.2018.00205

**Published:** 2018-07-20

**Authors:** Ma. G. Aguilera-Arreola, Martha L. Ostria-Hernández, Enrique Albarrán-Fernández, Sara R. Juárez-Enriquez, Cristina Majalca-Martínez, Beatríz Rico-Verdín, Enrico A. Ruiz, María del Socorro Ruiz-Palma, María R. Morales-García, Araceli Contreras-Rodríguez

**Affiliations:** ^1^Medical Bacteriology Laboratory, Department of Microbiology, Instituto Politécnico Nacional, Escuela Nacional de Ciencias Biológicas, Mexico City, Mexico; ^2^Department of Epidemiology, Centro Médico Nacional, 20 de Noviembre del Instituto de Seguridad y Servicios Sociales de los Trabajadores del Estado, Mexico City, Mexico; ^3^Special Test Laboratory, Centro Médico Nacional, 20 de Noviembre - Instituto de Seguridad y Servicios Sociales de los Trabajadores del Estado, Mexico City, Mexico; ^4^Ecology Laboratory, Department of Zoology, Instituto Politécnico Nacional, Escuela Nacional de Ciencias Biológicas, Mexico City, Mexico; ^5^General Microbiology Laboratory, Department of Microbiology, Instituto Politécnico Nacional, Escuela Nacional de Ciencias Biológicas, Mexico City, Mexico; ^6^Biotechnology Area, Instituto Politécnico Nacional, Centro de Investigación en Ciencia Aplicada y Tecnología Avanzada, Querétaro, Mexico

**Keywords:** *Ochrobactrum* bacteraemia, *Ochrobactrum* identification, misidentifying Brucella, *Ochrobactrum anthropi*, *Ochrobactrum* in humans

## Abstract

The present report describes the misidentification of *Brucella* spp. from a positive blood culture using traditional microbiology tests. A molecular test identified the bacterium as *Ochrobactrum anthropi*. According to the information available, this report is the first to include this type of case in Mexico.

## Background

Family *Brucellaceae* consists of six genera in addition to *Brucella* (*Ochrobactrum, Crabtreella, Daeguia, Mycoplana, Paenochrobactrum*, and *Pseudochrobactrum*). *Ochrobactrum* is an alphaproteobacterium belonging to the Rhizobiales order (1). Six species of *Ochrobactrum* have been described based on 16S rDNA sequencing (*O. anthropi, O. intermedium, O. tritici, O. grignonense, O. gallinifaecis*, and *O. lupine*) (2).

*Ochrobactrum anthropi* is a gram-negative bacillus that is non-fermenting, an obligate aerobe, flagellate, oxidase-positive, and indole-negative. *Ochrobactrum* spp. thrive in the rhizosphere and do not multiply in host cells. They exhibit low virulence and seldom cause human infections (1). *Ochrobactrum anthropi* is found in soil and water but can also be isolated from contaminated biological products, such as human wastes, fluids and medical devices (3, 4). *Ochrobactrum anthropi* has been described as an opportunistic pathogen that causes infections in severely-ill or immunocompromised patients through the use of indwelling catheterization, which can commonly lead to clinical manifestations such as catheter-related bloodstream infections (5–7).

In the last decade, *O. anthropi* has been associated to bacteraemia, brain empyema, endophthalmitis, septic shock, septic arthritis, endocarditis, and retropharyngeal abscess. Furthermore, this bacterium has caused several hospital outbreaks and in recent years, there have been reports of multi-drug resistant clinical isolates of *O. anthropi* (3, 4, 6–10). Therefore, treatment of *O. anthropi* infections should consider the type of infection as well as the susceptibility of the strain (7). Microbiological characterization of this pathogen is difficult due to its phenotypic similarities with other microorganisms, leading to potential mistakes in its diagnosis (11).

Based on molecular markers and genome comparisons, the *Brucella* genus is closest to the *Ochrobactrum genus* (12).

In this study, a case report involving the misidentification of *O. anthropi* as *Brucella* using traditional microbiology methods in an immunocompromised patient is described. *Ochrobactrum anthropi* was correctly identified by 16S ribosomal gene sequencing. The patient gave her written consent for the publication of this case report.

## Case report

A thirty-two-year-old female patient from Mexico City was hospitalized in the National Medical Centre 20 of November-ISSSTE, which is a tertiary care hospital in Mexico City, due to general discomfort and a background of lymphoblastic leukemia, allogeneic bone marrow transplant, and cervical cancer. Five days before hospital admission, she displayed shooting cephalea, night sweats, asthenia, adynamia, dyspnoea, and a fever (39°C) of unknown origin that occurred primarily at night. Additionally, she showed transvaginal bleeding, which led her to seek treatment. When she was admitted into the unit, she exhibited haematomas in her arms and legs; a clinical report showed a leukocyte count of 1,800 cells per cubic millimeter, 4.5% neutrophils, 8.5 g/dL hemoglobin, 24.5% haematocrit and a platelet count of 11,000 cells/mm^3^. During the first day in the hospital, the patient received a blood transfusion and presented additional complications unrelated to the infection. A fever of 39°C and fatigue in addition to sweating were recorded for 3 days, but no sign of an infection focus could be identified. Despite the aforementioned, the patient was still treated with imipenem (500 mg IV/6 h) and ciprofloxacin (500 mg/8 h) for 16 days and showed no improvement. A chest X-ray was performed to check for the presence of a pneumonic focus or injuries; however, no sign of infection could be detected. Being that the fever (39°C) persisted after the previously described treatment, 20 mL of venous blood was taken from a central venous catheter and inoculated into two aerobic blood culture bottles. A gram-negative bacterium was isolated from the central catheter, but the automated Vitek 2® system was not able to identify it. Then, a new treatment was given to the patient consisting of amikacin (1 g/24 h for 15 days), ceftazidime (2 g/8 h for 15 days), caspofungin (500 mg/24 h for 15 days), and normal human immunoglobulin. Ten days after completing this second treatment, 20 mL of blood was taken from a peripheral vein and cultured in two bottles; nevertheless, both cultures were negative.

Because fever (39°C) and fatigue together with sweating reappeared, two haemocultures from a peripheral vein were performed 20 days after taking the second treatment. The blood culture bottles tested positive for gram-negative rods after a 24-h incubation, and the bacterium grew on a GC agar base and sheep blood medium. The strain could not be reliably identified using the Vitek 2® automated equipment, but the symptoms (primarily night fever and sweats) suggested the possibility of an infection caused by *Brucella* spp.

**Figure 1:**
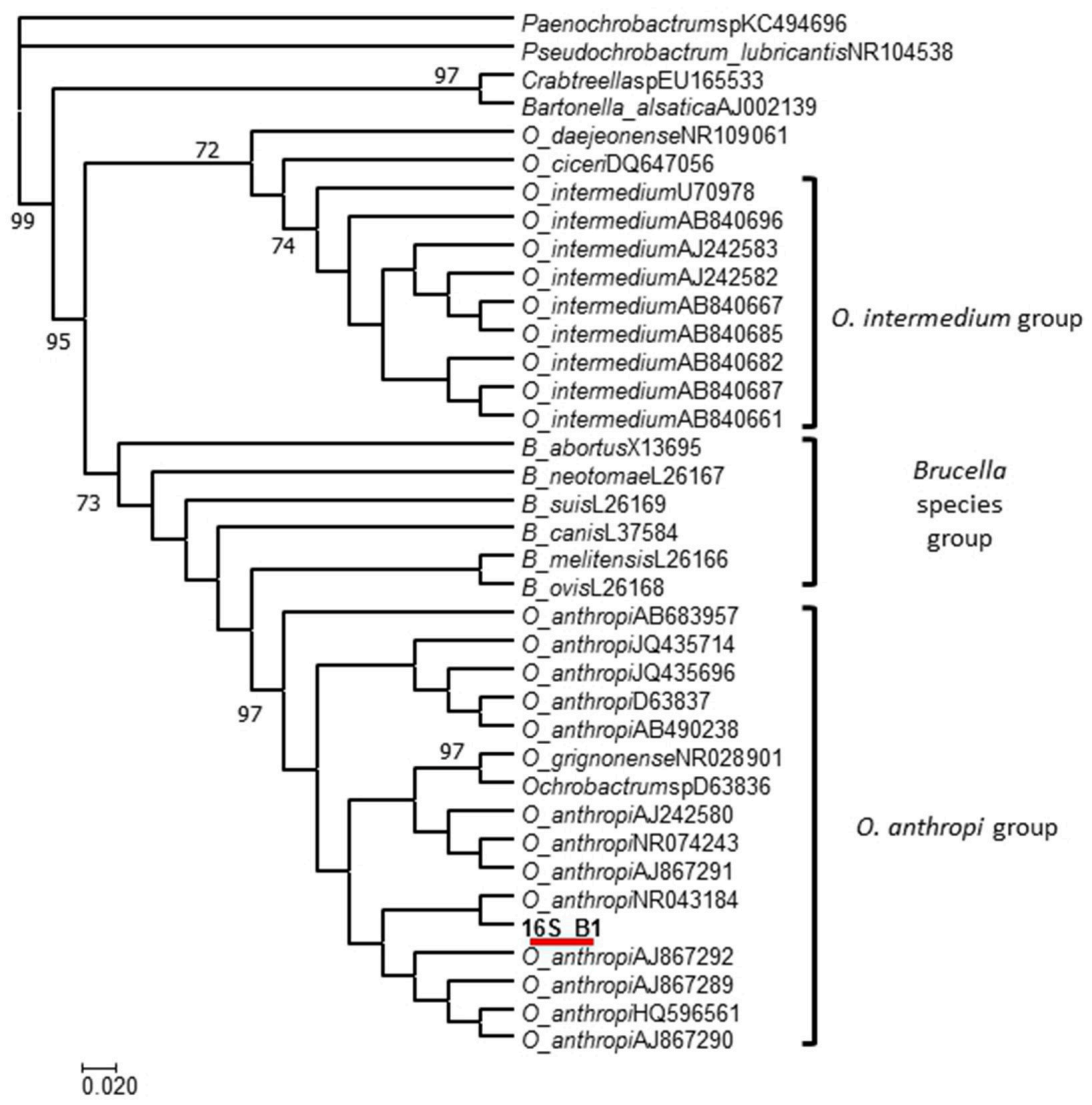
ML phylogeny of the *O. anthropi* 16S sequences deposited in GenBank. The results revealed that our 16S sequence 16S_B1 has the closest phylogenetic relationship with *O. anthropi*.

The strain was sent to the Microbiology Department at the National School of Biological Sciences, National Polytechnic Institute (IPN) in Mexico City for proper identification. The isolated bacterium was tested with polyvalent antisera against *Brucella*, and a positive agglutination reaction was observed. Based on this result, a molecular approach to confirm the presence of *Brucella* spp. was taken. Hence, DNA was obtained employing the DNAzol® reagent following the Thermo Fisher Scientific's protocol. An end-point polymerase chain reaction (PCR) was performed to amplify a specific sequence of the *bscp31* gene (223 bp), which is specific to *Brucella* as previously described by Baily et al. (13). Given the positive results, we proceeded to identify the species following a multiplex PCR assay as previously reported by García-Yoldi et al. (14). The results of this test were negative, indicating that the isolated bacterium did not belong to the *Brucella* species. These contradicting results called for further testing, therefore a broad-range PCR reaction focused on the amplification of a segment of the 16S ribosomal gene (510 bp) was performed following the conditions described by Aguilera-Arreola et al. (15). The PCR product was sequenced, and the 16S assembled sequence (16S_B1) was uploaded into GenBank under accession number KY982960. A maximum likelihood (ML) analysis was performed in order to test the phylogenetic relationship between the 16S consensus sequence of the strain against a set of the most similar *Ochrobactrum* 16S sequences found in the GenBank. The species and the corresponding GenBank accession numbers of the sequences used in this ML analysis were as follows: *Ochrobactrum* sp. (D63836), *O. anthropi* (D63837, NR074243, NR043184, JQ435696, JQ435714, AB683957, HQ596561, AB490238, AJ867292, AJ867292, AJ867290, AJ867289, and AJ242580), *O. intermedium* (U70978, AJ242583, AJ242582, AB840685, AB840661, AB840696, AB840682, AB840667, and AB840687), *O. ciceri* (DQ647056), *O. daejonense* (NR109061), *O. grignonense* (NR028901), *B. abortus* (X13695), *B. ovis* (L26168), *B. melitensis* (L26166), *B. neotomae* (L26167), *B. suis* (L26169), *B. canis* (L37584), *Pseudochrobactrum lubricantis* (NR104538), *Paenochrobactrum* sp. (KC494696), *Crabtreella* sp. (EU165533), and *Bartonella alsatica* (AJ002139). To perform the ML method, an algorithm in the aLRT-PHYML programme (16) in conjunction with an optimized base frequency and an estimated transition/transversion ratio were used. Optimization of the tree topology and branch length were obtained, and the confidence of each node was estimated via the approximate likelihood ratio test (aLRT) (17) using the Shimodaira-Hasegawa-like option. The *Bartonella alsatica* 16S sequence was included as an outgroup. The resulting ML phylogeny revealed that the 16S sequence of the tested bacterium (16S_B1) had the closest phylogenetic relationship with the *O. anthropi* 16S sequences deposited in GenBank. This relationship was revealed when the location of the 16S sequence obtained was found within a clade containing exclusively *O. anthropi* 16S sequences (aLRT >70; Figure 1). Conversely, this sequence was not located within the clade containing *O. intermedium* 16S sequences.

The susceptibility of the strain to 19 antimicrobial agents was tested by the Kirby-Bauer method as established by the Clinical Laboratory Standards Institute and considering suggestions for *Haemophilus* spp. and gram-negative bacteria. The strain was resistant to ampicillin, cefazoline, ceftazidime, cefoxitin, ceftriaxone, cefepime, aztreonam, nitrofurantoin, ampicillin/sulbactam, amoxicillin/clavulanic acid, piperacillin/tazobactam, meropenem, and tetracycline. The strain showed susceptibility to trimethoprim/sulfamethoxazole, ertapenem, amikacin, gentamicin, and ciprofloxacin. Based on the sensitivity of the isolated strain to these antibiotics, the chosen treatment for the patient was amikacin (1 g/24 h) and ciprofloxacin (500 mg/8 h) for 31 days. The patient completed the treatment and responded favorably to the combination of antibiotics; at the end of the treatment, the fever had disappeared, and the patient was released from the hospital. Three months later, the patient came back into the hospital for a medical check-up, but no symptoms related to the former infection were recorded.

These findings highlight the importance of *O. anthropi* as a nosocomial pathogen (even when it displays few virulence factors) and the importance of using more than one method to identify this uncommon pathogen.

## Discussion

The accurate identification of microorganisms involved in infectious diseases is paramount for clinical microbiology, since it can lead to specific and effective treatments and help prevent antibiotic resistance (15).

Based on DNA, rRNA and protein analyses, *O. anthropi* is one of the closest relatives of *Brucella*. The two genera share phenotypic characteristics (i.e., non-fermenting obligate aerobe metabolism, gram-negative rods and both are positive to the catalase and urease test) (1). These common features lead to identification problems. In this work, the gram-negative bacterium isolated from the haemocultures was able to induce positive agglutination using anti-*Brucella* serum. *Ochrobactrum anthropi* and *Brucella* spp. have similar molecules in their outer membranes, including phosphatidylcholine and a lipopolysaccharide (LPS) with a lipid A that consists of long-chain fatty acids. Nonetheless, *O. anthropi* and *Brucella* differ in the chemical structures of their lipid A cores. Therefore, the observed agglutination reaction may have yielded a positive result due to recognition of epitopes found in both bacterial envelopes. Moreover, both *Brucella* spp. and *O. anthropi* provoke fever as a general symptom but show no other clinical manifestations. Because of their particular metabolic characteristics, the analyst should be cautious when identifying these strains using semiautomatic and automatic biochemical test systems (i.e., the RapID NF Plus and VITEK 2) being that misidentification has previously been reported (1). Other authors have previously mentioned that *anti-Brucella* sera may cause an agglutination reaction when exposed to other bacteria, including *Yersinia enterocolitica* O:9, *E. coli* O:157, *O. anthropi* and *Afipia clevelandensis* (18–20). The cross reaction is relevant because the diagnostic methods used for human brucellosis are mainly based on serological tests. After taking into account the positive agglutination reaction result of the strain assayed in this study, the bacterium was mistakenly identified as *Brucella* spp. Thus, the strain was further tested using Brucella-genus-specific PCR. The *bscp31* PCR results showed positive amplification of the expected product, indicating that the strain was *Brucella*. At this point, we attempted to identify the species using a multiplex PCR method that is able to identify 6 *Brucella* species, including 2 vaccine strains, but no amplification was observed.

Over the last two decades, many efforts have been made to identify bacteria through the use of DNA fingerprinting methods (21). One of the most commonly used methods to identify bacteria is 16S rRNA gene sequencing. Universal primers designed to amplify the hypervariable V1, V2 and V3 regions of the 16S rRNA gene have been used to identify uncommon clinical bacteria as well as environmental strains that are not included in the databases of automated systems (15). Following this method, proper analysis of the sequences can lead to better identification of many organisms, including several that have rarely been studied (22). In this work, a broad-range PCR that includes the V1-V3 regions of 16S rRNA (15) was useful to discriminate between *Brucella* and *Ochrobactrum* despite their close phylogenetic relationship. When the presence of *Brucella* is suspected in a blood culture, the recommendation is to avoid manipulation of the bacterium outside of a class 3 laboratory. In Mexico, most public hospitals lack this type of facility; however, the National Reference Institute has a class 3 laboratory and a few research academic facilities where brucella can be identified. Another alternative that has been used over the past several years for the rapid identification of bacteria is matrix-assisted laser desorption/ionization-time of flight (MALDI-TOF) mass spectrometry, which decreases handling of the cultures and can assist with identification, especially in the case of *Brucella* (23).

The challenge for automated systems based on metabolic tests is the need to extend the databases in order to include less common bacteria, which would lead to the identification of metabolically inactive bacteria, such as the *Ochrobactrum* species. Conversely, developing a multiplex PCR including specific genes for *Brucella*, and *Ochrobactrum* at a genus level could also be a useful tool, since it is easy to perform in clinical laboratories.

Strains of *O*. *anthropi* have inherent resistance to beta-lactams. This resistance makes empirical treatment difficult because these antibiotics are commonly used against gram-negative bacteria. Teyssier et al. determined that clinical strains of *O. anthropi* were susceptible to gentamicin, tobramycin, rifampin, fluoroquinolones, netilmicin, colistin, and trimethoprim-sulfamethoxazole *in vitro* (2).

Cieslak et al. reviewed the treatments used in different clinical cases caused by *O. anthropi* and found that a combination of trimetroprim-sulfametoxazol and gentamicin or amikacin were useful in patients with bacteraemia (24). Other alternatives used in recent years to treat infections caused by *O. anthropi* have included quinolones, such as ciprofloxacin (25).

Because *O. anthropi* can adhere to catheters or other medical devices it has been related to catheter-related bacteraemia. Alnor et al. documented that *Agrobacterium* and *O. anthropi* infections in Danish patients were not eliminated by the antibiotic treatment itself, but until the catheters were removed (26).

The *O. anthropi* strain tested in this study exhibited a resistance pattern that had previously been reported in other human cases. However, because this bacterium is multi-drug resistant, a thoughtful selection of antimicrobial therapy, based on the susceptibility of the strain is necessary for successful treatment. In this case, the combination of ciprofloxacin and amikacin for 31 days was effective in eliminating the bacteria. In a case of osteomyelitis caused by *O. anthropi* in young men, 6 weeks of treatment with ciprofloxacin and clindamycin was successful. Unfortunately, at present, there is no specific treatment to manage *O. anthropi* infections.

This finding is relevant for hospitals in Mexico because it shows that *O. anthropi* could cause bacteraemia in immunocompromised patients as has previously been reported in other countries.

Unfortunately, there is currently no established procedure to identify *O. anthropi*. The challenge of overcoming technical difficulties in order to identify this bacterium is crucial. Thus, it is also important to share this case to give information as to how this kind of cases in Mexican public hospitals should be managed. Based on the present case report, whenever the automated metabolic identification fails, the use of MALDI-TOF protocol is recommended, because the identification is based on the analysis of ribosomal proteins. However, in this case report the sequencing of the 16SrDNA gene of the isolated bacterium was necessary to confirm the correct identification. Ultimately, when an isolate comes from a clinical case exhibiting fever of unknown origin and its identification using biochemical tests proves uncertain, molecular identification should be performed. In case the laboratory lacks the methodology or the equipment to perform the recommended protocols, the isolated bacterium should be sent to a reference laboratory confirming identification.

## Concluding remarks

The present work reports the first case of *O. anthropi* causing bacteraemia in Mexico. Because this species can easily be misidentified as *Brucella* spp., the identification protocols must be reviewed and enhanced. Furthermore, since several cases of infections caused by *Ochrobactrum* have been reported, the role of *O. anthropi* as an opportunistic pathogen should be considered, especially in cases where symptoms are nonspecific or when other common pathogens could not be identified. Reporting infections caused by *O. anthropi* will contribute to a better understanding of its pathogenesis, improve patient management and treatment as well as prevent antibiotic resistance.

## Ethics statement

The present work was approved by the ethics committee of the Centro Médico Nacional 20 de Noviembre (approval number 02/09 - 055.2009) in conjunction with the Instituto de Seguridad y Servicios Sociales de los Trabajadores del Estado, ISSSTE, Mexico City. Oral, and written informed consent was provided by the participant.

## Author contributions

MA-A, MO-H, MM-G, and AC-R performed all analyses and drafted the manuscript. ER performed the *in silico* analysis. SJ-E, CM-M, EA-F, and BR-V participated in the selection and collection of samples, processed the samples, obtained the permissions for the CMN 20 Nov and compiled all medical and clinical records from the patient. MA-A and AC-R planned the present study. All authors read, corrected, and approved the final manuscript.

### Conflict of interest statement

The authors declare that the research was conducted in the absence of any commercial or financial relationships that could be construed as a potential conflict of interest. The reviewer DP and the handling Editor declared their shared affiliation.
